# Bis{2-[3-(dimethyl­amino)­propyl­imino­meth­yl]-6-meth­oxy­phenolato}-κ^3^ *N*,*N*′,*O* ^1^;κ^2^ *N*,*O* ^1^-zinc(II) dihydrate

**DOI:** 10.1107/S1600536810052438

**Published:** 2010-12-18

**Authors:** Hong Lin, Xiao-Juan Wang

**Affiliations:** a Jinhua Professional–Technical College, Jinhua, Zhejiang 321007, People’s Republic of China; b Zhejiang Key Laboratory for Reactive Chemistry on Solid Surfaces, Institute of Physical Chemistry, Zhejiang Normal University, Jinhua, Zhejiang 321004, People’s Republic of China

## Abstract

In the title mononuclear Zn^II^ complex, [Zn(C_13_H_19_N_2_O_2_)_2_]·2H_2_O, the Zn^II^ atom is coordinated by two O atoms and three N atoms from two crystallographically different Schiff base ligands in a distorted trigonal–bipyramidal environment. One O and two N atoms constitute the base of the pyramid, and one O and one N atoms occupy the apical positions. Inter­molecular O—H⋯O and O—H⋯N hydrogen bonds between the lattice water mol­ecules and N/O atoms of the Schiff base ligands stabilize the conformation, whereas inter­molecular O—H⋯O hydrogen bonds between the two lattice water mol­ecules lead to a chain structure in [001].

## Related literature

For related structures, see: Choudhury *et al.* (2001 ▶); Guo & Lin (2008 ▶); Lin *et al.* (2009 ▶). 
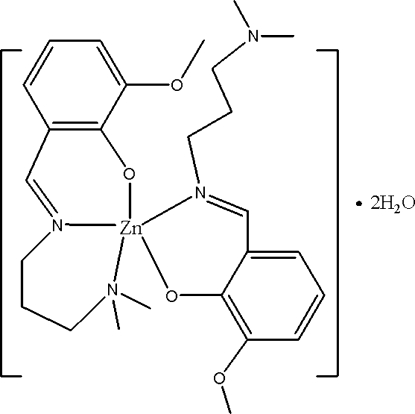


## Experimental

### 

#### Crystal data

[Zn(C_13_H_19_N_2_O_2_)_2_]·2H_2_O
*M* *_r_* = 572.01
Orthorhombic, 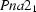

*a* = 14.982 (3) Å
*b* = 9.4411 (19) Å
*c* = 20.384 (4) Å
*V* = 2883.2 (10) Å^3^
*Z* = 4
Mo *K*α radiation
μ = 0.90 mm^−1^
*T* = 293 K
0.33 × 0.24 × 0.09 mm

#### Data collection

Bruker APEXII area-detector diffractometer
Absorption correction: multi-scan (*SADABS*; Sheldrick, 1996 ▶) *T*_min_ = 0.773, *T*_max_ = 0.923
14801 measured reflections
6371 independent reflections
4666 reflections with *I* > 2σ(*I*)
*R*_int_ = 0.027

#### Refinement

*R*[*F* ^2^ > 2σ(*F* ^2^)] = 0.031
*wR*(*F* ^2^) = 0.072
*S* = 1.00
6371 reflections
352 parameters
7 restraints
H atoms treated by a mixture of independent and constrained refinement
Δρ_max_ = 0.23 e Å^−3^
Δρ_min_ = −0.30 e Å^−3^
Absolute structure: Flack (1983 ▶), 2935 Friedel pairs
Flack parameter: −0.002 (11)

### 

Data collection: *APEX2* (Bruker, 2002 ▶); cell refinement: *SAINT* (Bruker, 2002 ▶); data reduction: *SAINT*; program(s) used to solve structure: *SHELXS97* (Sheldrick, 2008 ▶); program(s) used to refine structure: *SHELXL97* (Sheldrick, 2008 ▶); molecular graphics: *SHELXTL* (Sheldrick, 2008 ▶); software used to prepare material for publication: *SHELXTL*.

## Supplementary Material

Crystal structure: contains datablocks I, global. DOI: 10.1107/S1600536810052438/bq2261sup1.cif

Structure factors: contains datablocks I. DOI: 10.1107/S1600536810052438/bq2261Isup2.hkl

Additional supplementary materials:  crystallographic information; 3D view; checkCIF report

## Figures and Tables

**Table 1:** Hydrogen-bond geometry (Å, °)

*D*—H⋯*A*	*D*—H	H⋯*A*	*D*⋯*A*	*D*—H⋯*A*
O1*W*—H1*WA*⋯O3	0.82 (2)	2.02 (2)	2.805 (3)	161 (3)
O1*W*—H1*WA*⋯O4	0.82 (2)	2.49 (3)	3.067 (3)	129 (3)
O1*W*—H1*WB*⋯O1	0.83 (2)	2.39 (3)	3.019 (3)	133 (3)
O1*W*—H1*WB*⋯O2	0.83 (2)	2.58 (2)	3.379 (3)	162 (3)
O2*W*—H2*WA*⋯N4	0.85 (2)	2.05 (2)	2.894 (4)	178 (4)
O2*W*—H2*WB*⋯O1*W*^i^	0.84 (2)	2.06 (2)	2.900 (3)	175 (5)

Symmetry code: (i) 
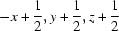
.

**Fig. 1.**
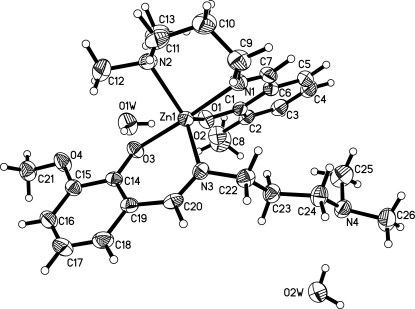
The molecular structure of (I), showing the atom-numbering scheme. Displacement ellipsoids are drawn at the 30% probability level.

**Fig. 2.**
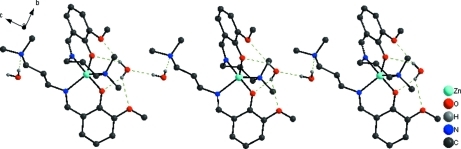
A view of the one-dimensional chain structure of (I). The hydrogen bond interactions are depicted by dashed lines.

## supplementary crystallographic information

### Comment

Transition metal complexes with multidentate Schiff base ligands have been extensively studied recently for their various crystallographic features, enzymatic reactions, catalysis, electrochemical and magnetic properties. Metal complexes with tridentate N_2_O Schiff base ligands derived from salicylaldehyde have been well studied in the past, such as [NiMe_2_NCH_2_CH_2_CH_2_N=CHC_6_H_4_O)_2_] (Choudhury *et al.*, 2001), [Ni(C_13_H_19_N_2_O_2_]_2_] (Guo *et al.*, 2008) and [Cd(C_13_H_20_N_2_O_2_)_2_*X*_2_] (Lin *et al.*, 2009). Molecule of the title complex (I) (Fig. 1) comprises one zinc(II) ion, two 2-[3-(dimethylamino)propyliminomethyl]-6-methoxyphenolato anions and two lattice water molecules. The center Zn^II^ atom is coordinated by two O atoms and three N atoms from two different Schiff base ligands in a distorted trigonal dipyramidal environment. Three coordinated atoms of O(1), N(2), and N(3) constitute the base of the pyramid, whereas N(1) and O(3) atoms occupy the apical position. The O and N atoms together with lattice water molecules are involved in hydrogen-bonding interactions (Fig.2). In detail, the structure is stabilized by intramolecular O—H···O and O—H···N hydrogen bonds between the lattice water molecules and N and O atoms from Schiff base ligands. The other intermolecular O—H···O hydrogen bonds between two lattice water molecules lead to a one-dimensional chain structure running along *c* direction.

### Experimental

3-methoxysalicylaldehyde (2.0 mmol) and 3-dimethylaminopropylamine (2.0 mmol) in 15 ml of methyl alcohol were stirred for 4 h. ZnSO_4_.7H_2_O (1.0 mmol) was added and stirred for 10 h. The resulting solution was placed in a refrigerator at 263 K for 7 days, and the crystals were filtered off, giving colorless cystals of the title complex for x-ray analysis.

### Refinement

The methyl groups were allowed to rotate to fit the electron density [O—H = 0.82 (2) Å, *U*_iso_(H) = 1.2*U*_eq_(O); C—H = 0.96 Å and *U*_iso_(H) = 1.5*U*_eq_(C)]; the other H atoms were positioned geometrically [aromatic C—H_aromatic_ 0.93 Å and aliphatic C—H = 0.97 Å, *U*_iso_(H) = 1.2*U*_eq_(C)].

### Crystal data

**Table:**

[Zn(C_13_H_19_N_2_O_2_)_2_]·2H_2_O	*F*(000) = 1216
*M**_r_* = 572.01	*D*_x_ = 1.318 Mg m^−^^3^
Orthorhombic, *P**n**a*2_1_	Mo *K*α radiation, λ = 0.71073 Å
Hall symbol: P 2c -2n	Cell parameters from 4118 reflections
*a* = 14.982 (3) Å	θ = 2.0–27.6°
*b* = 9.4411 (19) Å	µ = 0.90 mm^−^^1^
*c* = 20.384 (4) Å	*T* = 293 K
*V* = 2883.2 (10) Å^3^	Block, colorless
*Z* = 4	0.33 × 0.24 × 0.09 mm

### Data collection

**Table:**

Bruker APEXII area-detector diffractometer	6371 independent reflections
Radiation source: Bruker APEXII area-detector	4666 reflections with *I* > 2σ(*I*)
graphite	*R*_int_ = 0.027
ω scans	θ_max_ = 27.6°, θ_min_ = 2.0°
Absorption correction: multi-scan (*SADABS*; Sheldrick, 1996)	*h* = −19→19
*T*_min_ = 0.773, *T*_max_ = 0.923	*k* = −12→12
14801 measured reflections	*l* = −26→25

### Refinement

**Table:**

Refinement on *F*^2^	Secondary atom site location: difference Fourier map
Least-squares matrix: full	Hydrogen site location: inferred from neighbouring sites
*R*[*F*^2^ > 2σ(*F*^2^)] = 0.031	H atoms treated by a mixture of independent and constrained refinement
*wR*(*F*^2^) = 0.072	*w* = 1/[σ^2^(*F*_o_^2^) + (0.0255*P*)^2^] where *P* = (*F*_o_^2^ + 2*F*_c_^2^)/3
*S* = 1.00	(Δ/σ)_max_ = 0.001
6371 reflections	Δρ_max_ = 0.23 e Å^−^^3^
352 parameters	Δρ_min_ = −0.30 e Å^−^^3^
7 restraints	Absolute structure: Flack (1983), 2935 Friedel pairs
Primary atom site location: structure-invariant direct methods	Flack parameter: −0.002 (11)

### Special details

**Table:**

Geometry. All e.s.d.'s (except the e.s.d. in the dihedral angle between two l.s. planes) are estimated using the full covariance matrix. The cell e.s.d.'s are taken into account individually in the estimation of e.s.d.'s in distances, angles and torsion angles; correlations between e.s.d.'s in cell parameters are only used when they are defined by crystal symmetry. An approximate (isotropic) treatment of cell e.s.d.'s is used for estimating e.s.d.'s involving l.s. planes.
Refinement. Refinement of *F*^2^ against ALL reflections. The weighted *R*-factor *wR* and goodness of fit *S* are based on *F*^2^, conventional *R*-factors *R* are based on *F*, with *F* set to zero for negative *F*^2^. The threshold expression of *F*^2^ > σ(*F*^2^) is used only for calculating *R*-factors(gt) *etc*. and is not relevant to the choice of reflections for refinement. *R*-factors based on *F*^2^ are statistically about twice as large as those based on *F*, and *R*- factors based on ALL data will be even larger.

### Fractional atomic coordinates and isotropic or equivalent isotropic displacement parameters (Å^2^)

**Table:**

	*x*	*y*	*z*	*U*_iso_*/*U*_eq_	
Zn1	0.501709 (16)	0.23339 (2)	0.01203 (3)	0.04162 (7)	
O1	0.41420 (11)	0.08139 (17)	0.02808 (8)	0.0529 (4)	
O2	0.27116 (12)	−0.07155 (19)	0.03168 (10)	0.0629 (5)	
O3	0.43100 (11)	0.30686 (17)	−0.06314 (8)	0.0485 (4)	
O4	0.37783 (13)	0.34372 (19)	−0.18311 (9)	0.0654 (5)	
O1W	0.34773 (18)	0.0621 (2)	−0.11145 (11)	0.0825 (7)	
H1WA	0.373 (2)	0.139 (2)	−0.1067 (16)	0.099*	
H1WB	0.341 (2)	0.031 (3)	−0.0738 (11)	0.099*	
O2W	0.2026 (2)	0.4945 (4)	0.25478 (12)	0.1031 (8)	
H2WA	0.248 (2)	0.442 (4)	0.261 (2)	0.124*	
H2WB	0.185 (3)	0.511 (5)	0.2931 (11)	0.124*	
N1	0.58144 (13)	0.1529 (3)	0.08746 (12)	0.0537 (6)	
N2	0.61050 (15)	0.1672 (2)	−0.05394 (11)	0.0549 (6)	
N3	0.48338 (13)	0.4313 (2)	0.05770 (10)	0.0452 (5)	
N4	0.35581 (16)	0.3123 (3)	0.27371 (11)	0.0587 (6)	
C1	0.40909 (17)	−0.0067 (3)	0.07683 (12)	0.0438 (6)	
C2	0.33160 (18)	−0.0943 (3)	0.08146 (13)	0.0515 (6)	
C3	0.3217 (2)	−0.1901 (3)	0.13098 (16)	0.0625 (8)	
H3A	0.2708	−0.2465	0.1327	0.075*	
C4	0.3872 (3)	−0.2044 (3)	0.17916 (15)	0.0713 (9)	
H4A	0.3794	−0.2685	0.2133	0.086*	
C5	0.4618 (2)	−0.1250 (3)	0.17601 (14)	0.0642 (7)	
H5A	0.5057	−0.1370	0.2078	0.077*	
C6	0.47512 (18)	−0.0246 (3)	0.12599 (13)	0.0508 (6)	
C7	0.55804 (18)	0.0535 (3)	0.12662 (14)	0.0566 (7)	
H7A	0.5989	0.0290	0.1590	0.068*	
C8	0.1961 (2)	−0.1628 (4)	0.02810 (19)	0.0948 (12)	
H8A	0.1614	−0.1396	−0.0100	0.142*	
H8B	0.1602	−0.1512	0.0667	0.142*	
H8C	0.2160	−0.2592	0.0251	0.142*	
C9	0.67298 (18)	0.2078 (4)	0.09311 (17)	0.0738 (9)	
H9A	0.6996	0.1743	0.1336	0.089*	
H9B	0.6716	0.3105	0.0944	0.089*	
C10	0.7290 (2)	0.1591 (4)	0.03513 (18)	0.0797 (10)	
H10A	0.7902	0.1888	0.0424	0.096*	
H10B	0.7285	0.0564	0.0340	0.096*	
C11	0.6997 (2)	0.2131 (4)	−0.03087 (18)	0.0778 (10)	
H11A	0.7005	0.3158	−0.0296	0.093*	
H11B	0.7436	0.1836	−0.0631	0.093*	
C12	0.6000 (3)	0.2242 (4)	−0.12109 (19)	0.0991 (13)	
H12A	0.6495	0.1946	−0.1477	0.149*	
H12B	0.5982	0.3258	−0.1194	0.149*	
H12C	0.5454	0.1893	−0.1398	0.149*	
C13	0.6053 (2)	0.0128 (4)	−0.05818 (19)	0.0838 (10)	
H13A	0.6570	−0.0227	−0.0806	0.126*	
H13B	0.5525	−0.0136	−0.0819	0.126*	
H13C	0.6029	−0.0265	−0.0148	0.126*	
C14	0.41844 (15)	0.4378 (2)	−0.08135 (12)	0.0428 (6)	
C15	0.38774 (16)	0.4642 (3)	−0.14615 (13)	0.0482 (6)	
C16	0.37146 (18)	0.5993 (3)	−0.16748 (14)	0.0590 (7)	
H16A	0.3523	0.6140	−0.2103	0.071*	
C17	0.3831 (2)	0.7150 (3)	−0.12611 (17)	0.0689 (8)	
H17A	0.3711	0.8061	−0.1410	0.083*	
C18	0.41185 (19)	0.6940 (3)	−0.06417 (16)	0.0626 (7)	
H18A	0.4193	0.7715	−0.0365	0.075*	
C19	0.43089 (15)	0.5564 (3)	−0.04033 (12)	0.0458 (6)	
C20	0.45707 (16)	0.5433 (2)	0.02733 (12)	0.0484 (6)	
H20A	0.4547	0.6259	0.0521	0.058*	
C21	0.3374 (2)	0.3549 (3)	−0.24481 (13)	0.0692 (8)	
H21A	0.3276	0.2619	−0.2624	0.104*	
H21B	0.3757	0.4074	−0.2738	0.104*	
H21C	0.2813	0.4031	−0.2406	0.104*	
C22	0.49389 (17)	0.4456 (3)	0.12804 (13)	0.0541 (6)	
H22A	0.4984	0.5452	0.1394	0.065*	
H22B	0.5486	0.3992	0.1417	0.065*	
C23	0.41505 (17)	0.3800 (3)	0.16412 (13)	0.0567 (7)	
H23A	0.3615	0.4338	0.1544	0.068*	
H23B	0.4061	0.2839	0.1486	0.068*	
C24	0.42988 (19)	0.3780 (3)	0.23717 (13)	0.0615 (7)	
H24A	0.4844	0.3265	0.2465	0.074*	
H24B	0.4378	0.4745	0.2525	0.074*	
C25	0.3547 (3)	0.1609 (3)	0.26234 (18)	0.0878 (10)	
H25A	0.3086	0.1183	0.2883	0.132*	
H25B	0.4114	0.1213	0.2744	0.132*	
H25C	0.3435	0.1428	0.2167	0.132*	
C26	0.3640 (3)	0.3397 (4)	0.34359 (14)	0.0808 (10)	
H26A	0.3158	0.2944	0.3664	0.121*	
H26B	0.3617	0.4399	0.3513	0.121*	
H26C	0.4198	0.3028	0.3592	0.121*	

### Atomic displacement parameters (Å^2^)

**Table:**

	*U*^11^	*U*^22^	*U*^33^	*U*^12^	*U*^13^	*U*^23^
Zn1	0.03884 (11)	0.04110 (12)	0.04493 (13)	−0.00035 (12)	−0.00269 (13)	−0.0007 (2)
O1	0.0492 (9)	0.0547 (10)	0.0548 (12)	−0.0107 (8)	−0.0075 (8)	0.0098 (8)
O2	0.0537 (10)	0.0627 (12)	0.0724 (15)	−0.0166 (9)	−0.0013 (9)	0.0003 (9)
O3	0.0580 (10)	0.0357 (9)	0.0520 (10)	−0.0006 (7)	−0.0125 (8)	0.0000 (8)
O4	0.0898 (15)	0.0527 (11)	0.0537 (12)	0.0042 (10)	−0.0228 (10)	0.0007 (9)
O1W	0.122 (2)	0.0499 (12)	0.0752 (14)	−0.0135 (12)	−0.0216 (15)	−0.0070 (11)
O2W	0.123 (2)	0.120 (2)	0.0667 (16)	0.0474 (19)	−0.0175 (15)	−0.0036 (16)
N1	0.0387 (11)	0.0638 (15)	0.0586 (14)	0.0032 (11)	−0.0071 (10)	−0.0045 (12)
N2	0.0477 (13)	0.0566 (14)	0.0604 (15)	0.0065 (11)	0.0074 (10)	0.0005 (11)
N3	0.0466 (12)	0.0476 (12)	0.0413 (12)	−0.0035 (9)	0.0004 (9)	−0.0070 (9)
N4	0.0621 (15)	0.0655 (16)	0.0485 (14)	0.0026 (12)	0.0012 (11)	−0.0080 (11)
C1	0.0522 (15)	0.0361 (13)	0.0432 (14)	0.0042 (11)	0.0062 (11)	0.0000 (11)
C2	0.0595 (16)	0.0434 (14)	0.0516 (16)	0.0000 (12)	0.0161 (14)	−0.0049 (11)
C3	0.074 (2)	0.0469 (15)	0.067 (2)	−0.0033 (14)	0.0221 (16)	−0.0011 (14)
C4	0.105 (3)	0.0479 (18)	0.061 (2)	0.0131 (17)	0.0260 (19)	0.0135 (14)
C5	0.080 (2)	0.0602 (18)	0.0528 (18)	0.0214 (17)	0.0036 (16)	0.0060 (13)
C6	0.0585 (16)	0.0467 (14)	0.0471 (16)	0.0096 (12)	0.0038 (12)	0.0011 (11)
C7	0.0535 (17)	0.0614 (17)	0.0548 (18)	0.0142 (14)	−0.0101 (13)	−0.0011 (14)
C8	0.074 (2)	0.103 (3)	0.108 (3)	−0.040 (2)	0.001 (2)	−0.003 (2)
C9	0.0438 (16)	0.092 (2)	0.085 (2)	−0.0037 (16)	−0.0130 (16)	−0.0061 (18)
C10	0.0409 (15)	0.106 (3)	0.092 (3)	−0.0003 (17)	−0.0033 (15)	−0.003 (2)
C11	0.0478 (19)	0.091 (2)	0.095 (3)	−0.0036 (17)	0.0188 (18)	−0.0029 (19)
C12	0.090 (3)	0.139 (3)	0.069 (2)	0.024 (2)	0.022 (2)	0.023 (2)
C13	0.082 (2)	0.069 (2)	0.100 (3)	0.0112 (18)	0.016 (2)	−0.020 (2)
C14	0.0341 (12)	0.0416 (13)	0.0527 (16)	0.0018 (10)	0.0008 (11)	0.0009 (11)
C15	0.0449 (14)	0.0484 (15)	0.0512 (16)	0.0001 (11)	−0.0018 (12)	0.0025 (12)
C16	0.0545 (16)	0.0606 (18)	0.0617 (19)	0.0068 (14)	−0.0026 (13)	0.0146 (14)
C17	0.082 (2)	0.0446 (16)	0.080 (2)	0.0102 (15)	0.0018 (17)	0.0110 (15)
C18	0.0719 (19)	0.0419 (15)	0.074 (2)	0.0064 (13)	0.0077 (16)	−0.0069 (14)
C19	0.0390 (13)	0.0433 (14)	0.0552 (17)	0.0034 (11)	0.0044 (11)	−0.0001 (11)
C20	0.0443 (13)	0.0395 (12)	0.061 (2)	−0.0020 (11)	0.0094 (12)	−0.0129 (11)
C21	0.086 (2)	0.070 (2)	0.0521 (19)	−0.0025 (16)	−0.0141 (16)	−0.0014 (14)
C22	0.0529 (16)	0.0562 (15)	0.0532 (16)	−0.0028 (13)	−0.0050 (13)	−0.0116 (12)
C23	0.0539 (15)	0.0653 (18)	0.0509 (17)	−0.0032 (14)	−0.0054 (13)	−0.0067 (13)
C24	0.0697 (19)	0.0656 (18)	0.0491 (18)	−0.0109 (15)	−0.0047 (14)	−0.0066 (13)
C25	0.100 (3)	0.069 (2)	0.094 (3)	−0.0146 (19)	0.024 (2)	−0.0051 (19)
C26	0.099 (3)	0.090 (2)	0.053 (2)	−0.002 (2)	0.0011 (17)	0.0009 (16)

### Geometric parameters (Å, °)

**Table:**

Zn1—O1	1.9711 (16)	C9—H9B	0.9700
Zn1—O3	1.9878 (17)	C10—C11	1.504 (5)
Zn1—N1	2.090 (2)	C10—H10A	0.9700
Zn1—N3	2.106 (2)	C10—H10B	0.9700
Zn1—N2	2.204 (2)	C11—H11A	0.9700
O1—C1	1.298 (3)	C11—H11B	0.9700
O2—C2	1.377 (3)	C12—H12A	0.9600
O2—C8	1.418 (3)	C12—H12B	0.9600
O3—C14	1.304 (3)	C12—H12C	0.9600
O4—C15	1.372 (3)	C13—H13A	0.9600
O4—C21	1.400 (3)	C13—H13B	0.9600
O1W—H1WA	0.818 (17)	C13—H13C	0.9600
O1W—H1WB	0.826 (17)	C14—C19	1.410 (3)
O2W—H2WA	0.850 (18)	C14—C15	1.421 (3)
O2W—H2WB	0.838 (18)	C15—C16	1.370 (4)
N1—C7	1.281 (3)	C16—C17	1.390 (4)
N1—C9	1.471 (3)	C16—H16A	0.9300
N2—C13	1.462 (4)	C17—C18	1.349 (4)
N2—C12	1.479 (4)	C17—H17A	0.9300
N2—C11	1.482 (4)	C18—C19	1.415 (4)
N3—C20	1.287 (3)	C18—H18A	0.9300
N3—C22	1.449 (3)	C19—C20	1.439 (4)
N4—C25	1.448 (4)	C20—H20A	0.9300
N4—C26	1.453 (4)	C21—H21A	0.9600
N4—C24	1.473 (3)	C21—H21B	0.9600
C1—C6	1.418 (4)	C21—H21C	0.9600
C1—C2	1.428 (3)	C22—C23	1.523 (4)
C2—C3	1.364 (4)	C22—H22A	0.9700
C3—C4	1.395 (5)	C22—H22B	0.9700
C3—H3A	0.9300	C23—C24	1.506 (4)
C4—C5	1.348 (4)	C23—H23A	0.9700
C4—H4A	0.9300	C23—H23B	0.9700
C5—C6	1.406 (4)	C24—H24A	0.9700
C5—H5A	0.9300	C24—H24B	0.9700
C6—C7	1.445 (4)	C25—H25A	0.9600
C7—H7A	0.9300	C25—H25B	0.9600
C8—H8A	0.9600	C25—H25C	0.9600
C8—H8B	0.9600	C26—H26A	0.9600
C8—H8C	0.9600	C26—H26B	0.9600
C9—C10	1.521 (4)	C26—H26C	0.9600
C9—H9A	0.9700		
			
O1—Zn1—O3	91.58 (7)	N2—C11—H11B	108.1
O1—Zn1—N1	89.63 (8)	C10—C11—H11B	108.1
O3—Zn1—N1	176.90 (9)	H11A—C11—H11B	107.3
O1—Zn1—N3	119.07 (8)	N2—C12—H12A	109.5
O3—Zn1—N3	87.80 (7)	N2—C12—H12B	109.5
N1—Zn1—N3	94.12 (9)	H12A—C12—H12B	109.5
O1—Zn1—N2	112.75 (8)	N2—C12—H12C	109.5
O3—Zn1—N2	91.31 (8)	H12A—C12—H12C	109.5
N1—Zn1—N2	85.59 (9)	H12B—C12—H12C	109.5
N3—Zn1—N2	128.17 (8)	N2—C13—H13A	109.5
C1—O1—Zn1	129.26 (16)	N2—C13—H13B	109.5
C2—O2—C8	117.7 (2)	H13A—C13—H13B	109.5
C14—O3—Zn1	128.83 (15)	N2—C13—H13C	109.5
C15—O4—C21	118.5 (2)	H13A—C13—H13C	109.5
H1WA—O1W—H1WB	105 (2)	H13B—C13—H13C	109.5
H2WA—O2W—H2WB	103 (3)	O3—C14—C19	124.4 (2)
C7—N1—C9	117.7 (2)	O3—C14—C15	118.5 (2)
C7—N1—Zn1	124.63 (18)	C19—C14—C15	117.1 (2)
C9—N1—Zn1	117.6 (2)	C16—C15—O4	125.3 (2)
C13—N2—C12	107.6 (3)	C16—C15—C14	121.0 (2)
C13—N2—C11	111.0 (2)	O4—C15—C14	113.6 (2)
C12—N2—C11	106.5 (3)	C15—C16—C17	121.1 (3)
C13—N2—Zn1	106.22 (19)	C15—C16—H16A	119.5
C12—N2—Zn1	112.48 (19)	C17—C16—H16A	119.5
C11—N2—Zn1	112.99 (18)	C18—C17—C16	119.5 (3)
C20—N3—C22	115.6 (2)	C18—C17—H17A	120.3
C20—N3—Zn1	123.81 (17)	C16—C17—H17A	120.3
C22—N3—Zn1	120.40 (17)	C17—C18—C19	121.4 (3)
C25—N4—C26	109.5 (3)	C17—C18—H18A	119.3
C25—N4—C24	110.1 (2)	C19—C18—H18A	119.3
C26—N4—C24	110.9 (2)	C14—C19—C18	119.9 (2)
O1—C1—C6	125.2 (2)	C14—C19—C20	122.4 (2)
O1—C1—C2	118.0 (2)	C18—C19—C20	117.5 (2)
C6—C1—C2	116.8 (2)	N3—C20—C19	127.9 (2)
C3—C2—O2	125.2 (3)	N3—C20—H20A	116.0
C3—C2—C1	121.4 (3)	C19—C20—H20A	116.0
O2—C2—C1	113.3 (2)	O4—C21—H21A	109.5
C2—C3—C4	120.5 (3)	O4—C21—H21B	109.5
C2—C3—H3A	119.7	H21A—C21—H21B	109.5
C4—C3—H3A	119.7	O4—C21—H21C	109.5
C5—C4—C3	119.8 (3)	H21A—C21—H21C	109.5
C5—C4—H4A	120.1	H21B—C21—H21C	109.5
C3—C4—H4A	120.1	N3—C22—C23	110.8 (2)
C4—C5—C6	121.8 (3)	N3—C22—H22A	109.5
C4—C5—H5A	119.1	C23—C22—H22A	109.5
C6—C5—H5A	119.1	N3—C22—H22B	109.5
C5—C6—C1	119.6 (3)	C23—C22—H22B	109.5
C5—C6—C7	117.3 (3)	H22A—C22—H22B	108.1
C1—C6—C7	123.0 (2)	C24—C23—C22	111.6 (2)
N1—C7—C6	127.2 (2)	C24—C23—H23A	109.3
N1—C7—H7A	116.4	C22—C23—H23A	109.3
C6—C7—H7A	116.4	C24—C23—H23B	109.3
O2—C8—H8A	109.5	C22—C23—H23B	109.3
O2—C8—H8B	109.5	H23A—C23—H23B	108.0
H8A—C8—H8B	109.5	N4—C24—C23	113.2 (2)
O2—C8—H8C	109.5	N4—C24—H24A	108.9
H8A—C8—H8C	109.5	C23—C24—H24A	108.9
H8B—C8—H8C	109.5	N4—C24—H24B	108.9
N1—C9—C10	110.3 (3)	C23—C24—H24B	108.9
N1—C9—H9A	109.6	H24A—C24—H24B	107.7
C10—C9—H9A	109.6	N4—C25—H25A	109.5
N1—C9—H9B	109.6	N4—C25—H25B	109.5
C10—C9—H9B	109.6	H25A—C25—H25B	109.5
H9A—C9—H9B	108.1	N4—C25—H25C	109.5
C11—C10—C9	115.6 (3)	H25A—C25—H25C	109.5
C11—C10—H10A	108.4	H25B—C25—H25C	109.5
C9—C10—H10A	108.4	N4—C26—H26A	109.5
C11—C10—H10B	108.4	N4—C26—H26B	109.5
C9—C10—H10B	108.4	H26A—C26—H26B	109.5
H10A—C10—H10B	107.5	N4—C26—H26C	109.5
N2—C11—C10	116.6 (3)	H26A—C26—H26C	109.5
N2—C11—H11A	108.1	H26B—C26—H26C	109.5
C10—C11—H11A	108.1		
			
O3—Zn1—O1—C1	−171.2 (2)	C3—C4—C5—C6	1.5 (4)
N1—Zn1—O1—C1	11.7 (2)	C4—C5—C6—C1	−0.8 (4)
N3—Zn1—O1—C1	−82.8 (2)	C4—C5—C6—C7	−179.2 (3)
N2—Zn1—O1—C1	96.8 (2)	O1—C1—C6—C5	−179.4 (2)
O1—Zn1—O3—C14	143.2 (2)	C2—C1—C6—C5	−0.1 (3)
N1—Zn1—O3—C14	−103.9 (15)	O1—C1—C6—C7	−1.1 (4)
N3—Zn1—O3—C14	24.2 (2)	C2—C1—C6—C7	178.2 (2)
N2—Zn1—O3—C14	−103.9 (2)	C9—N1—C7—C6	−173.9 (3)
O1—Zn1—N1—C7	−7.8 (2)	Zn1—N1—C7—C6	1.8 (4)
O3—Zn1—N1—C7	−120.7 (14)	C5—C6—C7—N1	−176.9 (3)
N3—Zn1—N1—C7	111.3 (2)	C1—C6—C7—N1	4.8 (4)
N2—Zn1—N1—C7	−120.7 (2)	C7—N1—C9—C10	107.7 (3)
O1—Zn1—N1—C9	167.9 (2)	Zn1—N1—C9—C10	−68.3 (3)
O3—Zn1—N1—C9	55.0 (16)	N1—C9—C10—C11	63.9 (4)
N3—Zn1—N1—C9	−73.0 (2)	C13—N2—C11—C10	−59.0 (4)
N2—Zn1—N1—C9	55.0 (2)	C12—N2—C11—C10	−175.8 (3)
O1—Zn1—N2—C13	−13.9 (2)	Zn1—N2—C11—C10	60.2 (3)
O3—Zn1—N2—C13	−106.2 (2)	C9—C10—C11—N2	−63.8 (4)
N1—Zn1—N2—C13	73.8 (2)	Zn1—O3—C14—C19	−19.8 (3)
N3—Zn1—N2—C13	165.60 (19)	Zn1—O3—C14—C15	162.02 (17)
O1—Zn1—N2—C12	103.6 (2)	C21—O4—C15—C16	−8.6 (4)
O3—Zn1—N2—C12	11.3 (2)	C21—O4—C15—C14	172.1 (2)
N1—Zn1—N2—C12	−168.7 (2)	O3—C14—C15—C16	178.5 (2)
N3—Zn1—N2—C12	−76.9 (2)	C19—C14—C15—C16	0.2 (3)
O1—Zn1—N2—C11	−135.9 (2)	O3—C14—C15—O4	−2.1 (3)
O3—Zn1—N2—C11	131.9 (2)	C19—C14—C15—O4	179.6 (2)
N1—Zn1—N2—C11	−48.1 (2)	O4—C15—C16—C17	179.7 (3)
N3—Zn1—N2—C11	43.6 (2)	C14—C15—C16—C17	−1.0 (4)
O1—Zn1—N3—C20	−106.31 (19)	C15—C16—C17—C18	0.8 (5)
O3—Zn1—N3—C20	−15.73 (19)	C16—C17—C18—C19	0.3 (5)
N1—Zn1—N3—C20	161.8 (2)	O3—C14—C19—C18	−177.4 (2)
N2—Zn1—N3—C20	74.2 (2)	C15—C14—C19—C18	0.8 (3)
O1—Zn1—N3—C22	69.01 (18)	O3—C14—C19—C20	−1.6 (4)
O3—Zn1—N3—C22	159.59 (17)	C15—C14—C19—C20	176.6 (2)
N1—Zn1—N3—C22	−22.85 (18)	C17—C18—C19—C14	−1.0 (4)
N2—Zn1—N3—C22	−110.48 (18)	C17—C18—C19—C20	−177.0 (3)
Zn1—O1—C1—C6	−9.4 (4)	C22—N3—C20—C19	−172.0 (2)
Zn1—O1—C1—C2	171.35 (16)	Zn1—N3—C20—C19	3.6 (3)
C8—O2—C2—C3	−5.7 (4)	C14—C19—C20—N3	9.6 (4)
C8—O2—C2—C1	173.9 (2)	C18—C19—C20—N3	−174.6 (3)
O1—C1—C2—C3	179.5 (2)	C20—N3—C22—C23	102.0 (3)
C6—C1—C2—C3	0.2 (4)	Zn1—N3—C22—C23	−73.7 (2)
O1—C1—C2—O2	−0.1 (3)	N3—C22—C23—C24	173.1 (2)
C6—C1—C2—O2	−179.4 (2)	C25—N4—C24—C23	70.9 (3)
O2—C2—C3—C4	−179.9 (2)	C26—N4—C24—C23	−167.8 (3)
C1—C2—C3—C4	0.6 (4)	C22—C23—C24—N4	−178.7 (2)
C2—C3—C4—C5	−1.4 (4)		

### Hydrogen-bond geometry (Å, °)

**Table:**

*D*—H···*A*	*D*—H	H···*A*	*D*···*A*	*D*—H···*A*
O1W—H1WA···O3	0.82 (2)	2.02 (2)	2.805 (3)	161 (3)
O1W—H1WA···O4	0.82 (2)	2.49 (3)	3.067 (3)	129 (3)
O1W—H1WB···O1	0.83 (2)	2.39 (3)	3.019 (3)	133 (3)
O1W—H1WB···O2	0.83 (2)	2.58 (2)	3.379 (3)	162 (3)
O2W—H2WA···N4	0.85 (2)	2.05 (2)	2.894 (4)	178 (4)
O2W—H2WB···O1W^i^	0.84 (2)	2.06 (2)	2.900 (3)	175 (5)

Symmetry codes: (i) −*x*+1/2, *y*+1/2, *z*+1/2.
